# Cumulus Cell DNA Damage as an Index of Human Oocyte Competence

**DOI:** 10.1007/s43032-021-00817-7

**Published:** 2021-12-14

**Authors:** Alejandro Baratas, Jaime Gosálvez, Moises de la Casa, Silvia Camacho, Monica Dorado-Silva, Stephen D. Johnston, Rosa Roy

**Affiliations:** 1 Biology Department, Autonomous University of Madrid, Madrid, Spain; 2 GINEFIV, Assisted Reproduction Centre, Madrid, Spain; 3 GINEMED, Assisted Reproduction Centre, Sevilla, Spain; 4 School of Agriculture and Food Sciences, The University of Queensland, Brisbane, Australia

**Keywords:** Cumulus cells (CCs), DNA fragmentation, Embryo quality, IVF/ICSI, Oocyte quality, Biomarker

## Abstract

The determination of oocyte quality is crucial for achieving effective syngamy post-sperm injection and embryonic development. Cumulus cells (CCs) have been proposed as biomarkers of oocyte quality because of their close bio-dynamic relationship with the oocyte. To determine the quality of the oocyte, CCs were sampled during oocyte preparation for ICSI to determine a CC DNA fragmentation index (CCDFI) of each individual oocyte using a variant of the chromatin dispersion test. One hundred and thirty oocytes were selected and studied from two Spanish fertility clinics, 90 of which were fertilized and developed to embryos. Significant differences were found between the CCDFI of unfertilized and fertilized oocytes (*p* < .001) and between the CCDFI of embryos that were discarded and those that developed suitable for transfer or cryopreservation (*p* < *.*001). Oocyte quality was negatively correlated with CCDFI (Spearman’s rho =  − 0.45; *p* < *.*001). Receiver operator characteristics curves (ROC) suggested that a cut-off value of 24% CCDFI was able to discriminate the capacity of the gametes to result in syngamy with a sensitivity and specificity of 75.6% and 65%, respectively. This cut-off supports the application of CCDFI as potential index for the evaluation of the reproductive potential of oocytes prior to fertilization.

## Introduction

The use of morphological criteria for determining oocyte quality is regarded as controversial given its low predictive value with respect to embryo survival and pregnancy outcome [1, 2]. It is, therefore, imperative that we explore new biomarkers that might complement and/or help to improve oocyte assessment and selection, for oocyte quality appears to be one of the most limiting factors to the expansive application of ART [3].

Cumulus cells (CCs) are a unique sub-lineage of granulosa cells that surround the oocyte of an antral follicle and that play an essential role in metabolic and nutritional support of the oocyte, the control of oocyte meiosis, ovulation, oocyte developmental competence, and fertilization. An increasing body of evidence highlight the importance of cumulus gene expression for the positive health of the oocyte [4, 5]. Hence, assuming that the physiological and morphological quality of the CCs are reflective of the overall health of the oocyte and given that these cells will typically be discarded in preparing the oocyte for ICSI, appraisal of the CCs may serve as an index or indirect predictor for the overall assessment and selection of the oocyte [6]. The association between the cumulus oocyte complex and oocyte has been derived from the behavior of both cells during folliculogenesis in situ and from the development of oocytes in vitro. Several studies have reported that in the absence of granulosa cells and CCs, oocytes from primordial follicles are not able to grow [7, 8]; in addition, the co-culture of oocytes with cumulus cells from mature oocytes improved the maturation rates of these oocytes [9].

The dependent relationship of the oocyte and the cumulus cell is reinforced because of the existence of cellular communication via gap junctions between both type of cells [10]. The oocyte in itself has low capacity for metabolizing energetic molecules such as glucose [11], and the metabolic and energetic contribution mostly emerge from the CCs to the oocyte. CCs provide substantial glycolytic activity to transform glucose into pyruvate, which is the primary energy source used by the oocyte during its maturation [12]. In recent years, several studies have been carried out to identify biomarkers of CCs that might serve as prognostic tools of oocyte quality, especially at the level of transcriptome expression [13, 14].

Within this scenario, it seems logical that a fully functional state of the CCs would be directly related with the competence of the oocyte. In fact, apoptosis and DNA fragmentation at the CCs and oocyte competence, using techniques such TUNEL, have indicated a direct relationship [15, 16]. The aim of the present investigation was to assess the impact of the CC DNA quality on the competence of the oocyte associated to produce reproductive outcome at the level of fertilization and embryonic development. Our primary hypothesis was that CCs with elevated levels of fragmented DNA will be correlated with poor oocyte competence and early embryonic development.

## Materials and Methods

### Sample Collection

The CCs used in study were obtained during the normal preparation of the oocyte for the ICSI procedure and were obtained from two Spanish fertility clinics (GINEFIV, Madrid, and GINEMED, Sevilla). Informed consent for the use of the patient’s CCs was obtained from all couples. This study was approved by the Autonomus University of Madrid Instituional Review Board (CEI-106- 2075). All 25 couples included in the study were diagnosed as presenting with idiopathic infertility for which no specific female or male factor was identified. All couples presented to the respective clinic after 1 year of failed pregnancy following natural conception. The study cohort included couples attending the clinic for their first reproductive cycle. The women included in this study had a mean (± SD) age of 33.5 ± 1.3 years, were not obese, tobacco smokers, or regular consumers of alcohol. In addition, these women signed a declaration indicating that they had no clinical history of diabetes, high blood pressure, recent bacterial infections, genetic determined alterations, evidence of hydrosalpinx, or polycystic ovaries.

In preparation for ICSI, all couples received homogenous follicular stimulation using 150–200 IU of r-FSH (GONAL-F®, Merck Serono, Australia) and 75 IU of hMG (Menopur 75, Ferring Pharmaceuticals, West Drayton, UK). This treatment was followed by the administration of synthetic gonadotropin (0.25 mg of Orgalutran®, Organon Ltd, Ireland). Ovulation was induced with 250 µg recombinant human chorionic gonadotropin (Ovitrelle, Merck, The Netherlands). ICSI fertilization was performed using normozoospermic semen samples from the partner. After aspiration retrieval of the cumulus oophorus complex (COC), the CCs were stripped from the oocyte by gentle pipetting in a solution of hyaluronic acid 1X (Hy-ase 10X, Vitrolife, Göteborg, Sweden).

Given that the clinics who participated in the study were in different regions of Spain and in order to stock-pile the CCs for more efficient processing and evaluation at the Autonomous University of Madrid, the CCs were cryopreserved. Cryoprotection involved using a freezing solution and a standard straw freezing protocol (Cryoprotect II, Nidacon International Göteborg, Sweden). The CCs were stored in liquid nitrogen for approximately 1 week before being evaluated. For analysis, the CCs were thawed by rapid immersion of the straw containing the cells in a waterbath set at 37 °C for 3 min. The cells were then pelleted at 400* g* for 10 min and reconstituted with fresh RPMI media (PMI Media 1640; Life Technologies; Carlsbad, CA, USA) in preparation for fragmentation analysis. To ascertain that the cryopreservation and thawing procedures had adverse influence on CC DNA damage, a preliminary analysis comparing the DNA fragmentation of freshly collected and frozen-thawed CCs was conducted and no significant differences were observed (Wilcoxon test; *n* = 8; negative rank = 5; positive rank 4.33; *Z* =  − 0.577; *p* = 0.564). It is important to note that once the cells are thawed, they need to be processed quickly (within 1 h) for DNA quality visualization; otherwise, the cells and the DNA molecule may deteriorate. While we cryopreserved the CCs in this study for logistical purposes, from a clinical perspective, the assay is most likely to be used on freshly collected CCs from individual patients.

### Chromatin Dispersion Test

DNA fragmentation was assessed using a variant of the chromatin dispersion test (D3-Max Kit; Halotech DNA, Madrid, Spain; [17]). Thawed cells were included in a microgel, treated for chromatin depletion, dehydrated in a graded series of ethanol baths, and stained with 4′,6-diamidino-2-fenilindol (DAPI; Sigma Aldrich, Madrid, Spain) diluted 1:1 with VectaShield antifade mounting medium (Vector, Laboratories, Burlingame, CA, USA) for fluorescence microscopy observation. A Nikon Eclipse microscope equipped with CoolLed illumination and equipped with a Nikon 12bits CCD (Nikon DS-Qi2; Nikon Instruments Inc., Tokyo, Japan) was used for analysis and image capture.

**Fig. 1:**
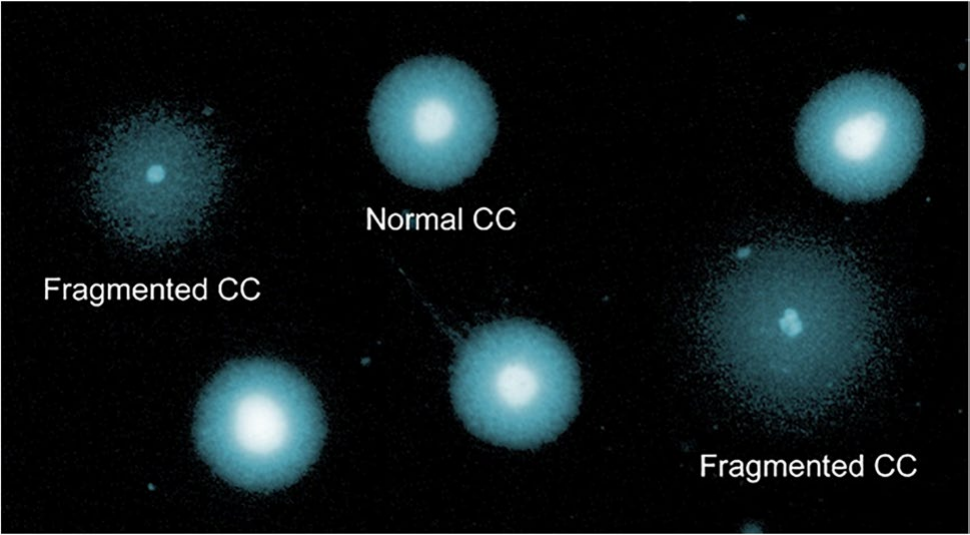
Visualization of DNA fragmentation in CCs using the chromatin dispersion assay

Two primary types of CCs were identified as previously described by Barcena et al. [17]. Cells presenting as a halo that were equivalent in size to the diameter of the nuclear core or less were considered as normal without DNA damage (Fig. 1). Cells with damaged DNA possessed halo diameters that were larger than their nuclear core and showed only faint fluorescence (Fig. 1). When the level of DNA fragmentation was extremely severe, the individual cell presented with a greatly reduced nuclear core diameter and only a residual halo (Fig. 1). A DNA fragmentation index (DFI) was established by calculating the ratio of the number of cells presenting with fragmented DNA with respect to the total number of cells scored on the slide.

### Experimental Design

DFI was analyzed with respect to oocyte and embryo quality. In analysis 1, the relationship between CCDFI and oocyte quality was categorized based on (1) oocytes that were recovered that were either immature or atretic (non-injected oocytes, NIO), (2) oocytes that failed to fertilize after ICSI (unfertilized oocytes, UFO), and (3) oocytes that were successfully fertilized following ICSI (fertilized oocytes, FEO). Data for studies of oocyte quality were supplied from both fertility clinics with 130 oocytes examined in total from 25 women. In analysis 2, in vitro embryo quality with respect to CCDFI was categorized based on whether or not the embryos were rejected following ICSI because of poor quality or developmental arrest after 1 day incubation (REJ) or whether they were suitable for either transfer or cryopreservation as assessed on day 3 or 5 of incubation (EMB). In analysis 3, the relationship between CCDFI and grade quality of the resulting fertilized embryo (designated A, B, and C using assessment criteria described by ASEBIR (Asociación para el Estudio de la Biología de la Reprodución) [18] was examined. Data for analysis 2 was based on 90 embryos whereas analysis 3 was based on 49 embryos.

Three ROC curves were also constructed. The first involved data from analysis 1 in which the ability of CC DFI to predict oocytes that failed to achieve syngamy (no syngamy = NIO + UFO) were compared to those that were fertilized (FEO). For the 2nd ROC curve, data from analysis 2 was used to determine the capacity of CCDFI to predict oocytes that were ultimately rejected by the embryologist (REJ) compared to those that were suitable for transfer and/or cryopreservation (EMB). The 3rd ROC curve examined how useful CCDFI was at predicting those oocytes following ICSI that developed to the stage where they were suitable for transfer or cryopreservation (EMB) compared those oocytes that failed to reach this status (REJ) and those that failed to achieve syngamy (NIO + UFO).

### Statistical Analysis

Statistical analysis was performed using the SPSS 25 statistics package (IBM, NY, USA). As data were not normally distributed, the Mann–Whitney *U* and Kruskal–Wallis tests were used to compare independent groups. Receiver operator characteristic (ROC) curves were conducted to test the predictive value of CCDFI with respect to oocyte and embryo quality. When determining predictive power, an area under the curve (AUC) of 0.7–0.8 was considered acceptable, whereas a value of 0.8–0.9 was considered excellent [19]. In all tests, statistical significance was set at a *p* < 0.05.

## Results

### Analysis 1: Distribution of CCDFI Within Stablished Groups

**Fig. 2:**
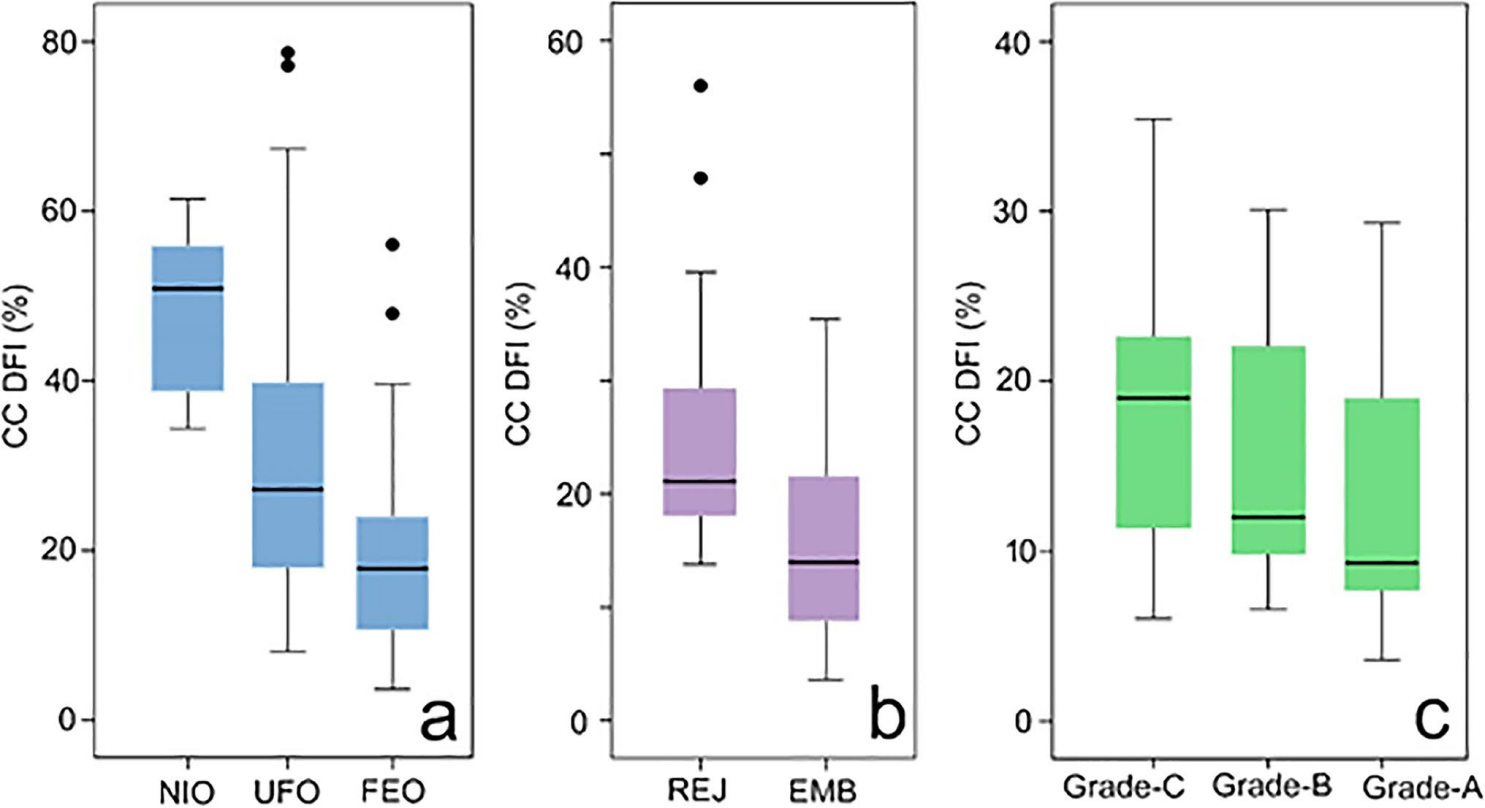
Box-Whisker plots of the CCDFI with respective to (**A**) increasing levels of oocyte quality (**B**) embryos rejected by the embryologist (REJ) and those incubated for 3–5 days (EMB) before transfer and (**C**) grade quality of the embryos produced based in morphological parameters

A total of 130 COC were retrieved from 25 patients and the results of the CCDFI of the different pre-stablished groups are shown in Fig. 2A. Six oocytes were discarded because the oocyte had not reached meiosis II (NIO) and the remaining 124 processed for ICSI. Thirty-four out of 124 failed to result in syngamy (UFO) leaving 90 of the oocytes showing evidence of successful fertilization after ICSI (FEO). A Kruskal–Wallis test revealed that CCDFI was significantly different over the 3 groups (H = 28.4, *p* < 0.001). Two independent sample comparisons between respective CCDFI categories based on an analysis with a Mann–Whitney *U* test were significantly different over the groups (*U* = 36, *p* = 0.012; *U* = 11, *p* < 0.001; *U* = 820, *p* < 0.001 between NIO-UFO, NIO-FEO, and UFO-FEO, respectively). Oocyte quality was negatively correlated with CCDFI (Spearman’s rho =  − 0.45; *p* < 0.001).

### Analysis 2: Embryo Development and CCDFI

With respect to embryo development, a total of 90 oocytes resulted in embryos that were incubated further, and the results of whether these embryos arrested and were then rejected by the embryologist or were suitable quality for transfer or cryopreservation are shown in Fig. 2b. A total of 31 FEO were rejected (REJ) after day 1 of incubation, whereas 59 embryos survived to day 3 or day 5 incubation (EMB) and were deemed suitable for transfer or cryopreservation. A Mann–Whitney test analysis of the CCDFI of the two cohorts showed a significant difference (*U* =  − 4.27; *p* < 0.001).

### Analysis 3: Embryo Grade and CCDFI

The relationship between the grade of embryos produced after 3 to 5 days of incubation and CCDFI was also examined, and the results reported in Fig. 2c. Of the embryos processed, a total of 49 were graded: 15 as A, 19 as B, and 15 as C grade. A Kruskal–Wallis test (H (2) = 2.19, *p* = 0.33) revealed that CCDFI was not significantly different over these 3 groups.

### Predicting Oocyte and Embryo Quality Using CCDFI

**Fig. 3:**
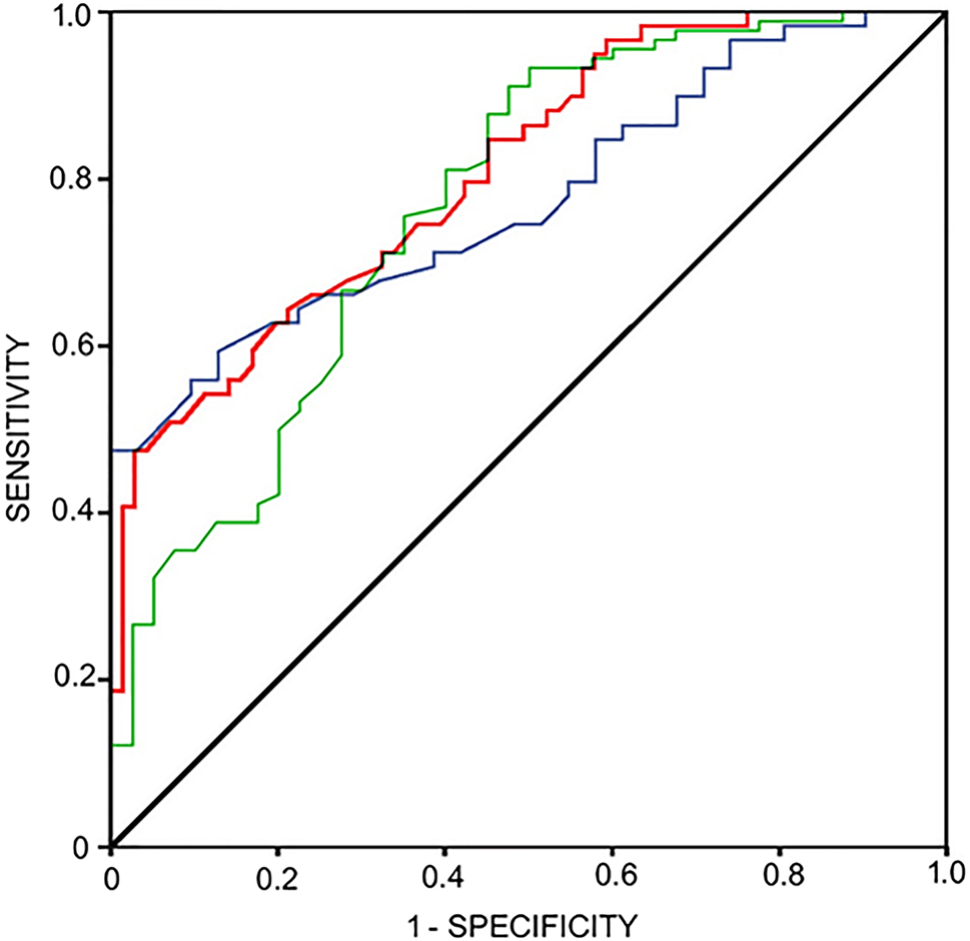
Green line ROC curve shows the ability of CCDFI to predict fertilized oocytes (FEO) and those that were not fertilized (NOI + UFO; AUC, .769; 95% CI [0.68–0.86]; SE, 0.046; *p* < .001). Blue line ROC shows the ability of CCDFI to predict embryos that were rejected by the embryologist (REJ) and those that were suitable for transfer and/or cryopreservation (AUC, .775; 95% CI [0.68–0.87]; SE, 0.048; *p* = .001). Red line ROC shows the ability of CCDFI to predict embryos reaching 3–5 days of development (AUC, 0.808; 95% CI [0.74–0.88]; SE, 0.037; *p* < .001)

ROC curves were used to assess for the sensitivity and specificity provided by the CCDFI. The first ROC curve compared UFO (*N* = 34) with oocytes that were successfully fertilized (FEO) (*N* = 90). For this analysis, the area under the curve was 0.769 (*p* < 0.001; 95% CI [0.68–0.86]; SE, 0.046). A threshold value of 24% CCDFI was able to discriminate the capacity of the gametes to result in syngamy with a 75.6% sensitivity and specificity of 65% (see green ROC curve of Fig. 3).

The second ROC curve was conducted to assess how the level of CCDFI-predicted embryo quality; this analysis compared the CCDFI of embryos rejected by the embryologist (REJ; *N* = 31) against those that were chosen for transfer or cryopreservation (EMB; *N* = 59). The area under the curve was 0.775 (*p* < 0.001; 95% CI [0.68–0.87]; SE, 0.048). The cut-off value for CCDFI was established at 20.3% with a sensitivity of 71.2% and a specificity of 61% (see blue ROC curve of Fig. 3).

The third analysis compared the CCDFI of embryos which successfully reached 3–5 days of development (*N* = 59) with those oocytes that did not reach this development following the ICSI procedure (*N* = 71). The area under the curve for this analysis was 0.808 (*p* < 0.001; 95% CI [0.74–0.88]; SE, 0.037) and the cut-off value for CCDFI was established at 21% with a sensitivity of 74.6% and a specificity of 64% (see red ROC curve of Fig. 3).

## Discussion

The results of this study suggest that oocytes with cumulus cells showing a high level of DNA damage have tendency to either immature, atretic, or correspond to oocytes associated fertilization failure after ICSI. In this investigation, we determined that a threshold value DFI of 24% was able to discriminate the capacity of the gametes to result in successful syngamy with a sensitivity of 75.6% and a specificity of 65%. Of particular interest was the fact that DFI was not a good predictor of embryo-grade quality after 3 to 5 days of incubation, as there were no significant differences in CCDFI between A, B, and C grade embryos. This observation highlights the issue that morphological criteria for best oocyte identification can be controversial due to its low predictive value with respect to embryo survival and pregnancy outcome [20].

In this study, we have found large differences in the level of DNA quality of CCs of different individual oocytes that may be concomitant with the results of fertilization and embryo quality observed at the first stages of development. In some animal models, apoptosis has been associated with the rate of follicular atresia observed in porcine [21], bovine [22], and rodent ovaries [23]. The ability of the oocyte to mature and fertilize has also been correlated with the apoptotic status of the cumulus cells [24]. Recently, Emanuelli et al. [25] have shown that bovine oocytes of greater quality exhibited lower levels of DNA fragmentation in their associated cumulus cells than oocytes of lower quality; these same authors also describe a negative correlation between nuclear fragmentation of CCs and the in vitro development of cattle embryos.

Conceptually, the male and female pronuclei have two roles or duties which can be negatively impacted by the presence of a damaged DNA molecule; these duties can be divided into tasks that occur before syngamy which can be referred to as their “haploid duty” and those that carry on after syngamy referred to as their “diploid duty.” Integrated with these individual gamete responsibilities, the situation is further complicated during syngamy, as it is now well described that the oocyte under certain circumstances has the capacity to repair the male DNA contribution [26], so that lack of normal oocyte maturation could potentially also compromise the oocyte’s DNA repair mechanisms. With respect to the oocyte’s haploid duties, histone H3/H4 acetylation pathways are key to achieving a nucleus ready for syngamy [27] and disequilibrium in the oolema has also been shown to prevent gamete fusion [5].

We need to acknowledge that other factors may also be impacting syngamy, such as the quality of the male gamete contribution. Under natural conception, once the sperm cell binds to the zona pellucida and penetrates into the perivitelline space of oocyte, a cascade of molecular changes enables the sperm nucleus to be engulfed into the ooplasm by the oolema, leading to the formation of the male pronucleus, DNA decondensation, culminating in syngamy. Binding of the sperm cell to the oolemma triggers an intracellular Ca^2+^ flux within the ooplasm [28] starting at the site of sperm penetration and pervading through the entire oocyte [29]. Given this study utilized ICSI, it is difficult to evaluate how the oocyte environment, in absence of this biological stimulus, may influence the outcome of syngamy but there are other haploid duties of the sperm required for successful syngamy. For example, gamete fusion is highly dependent on the maturation stage of the injected sperm cell as Kimura and Yanagimachi [30] have shown that while elongating spermatids have acquired the capacity of activating eggs, round spermatids are unable to induce activation. Some of these scenarios may help to explain arrested syngamy which was not insignificant in our experiment, as 27.4% of the oocytes failed to become fertilized after sperm injection. In this context, it is interesting to recall Albertini’s [31] suggestion “Syngamy has become an acceptable sentinel for the beginning of life.” Clearly, there is a need to gain better knowledge of the haploid duties of both the oocyte and sperm cell to better understand syngamy failure.

Once syngamy has been obtained, the oocyte is then occupied in fulfilling another role that we may refer to as its “diploid duty”; this involves control of the new pluripotent zygote to divide and generate into undifferentiated blastomeres until cell specialization occurs. This implies a series of epigenetic modifications of the genome including a series of hybrid or asymmetric patterns of histone methylation when paternal and maternal genomes are compared. For example, while H3 is methylated in the maternal genome, it is not methylated in the paternal genome, and this pattern is maintained during early embryonic development [32]. During this phase of development, any deregulation of the enzymes required to accomplish these processes, in combination with deficiencies associated with delivery of nutrients and hormones from the cumulus cells, may manifest in the arrest of embryonic development. With respect to the information relevant to the diploid duties (embryonic development) of the oocyte in the current study, we differentiated 2 groups, embryos discarded after 1 day incubation and embryos that successfully developed to stage (3–5 days) suitable for transfer and/or cryopreservation. We have demonstrated that oocytes reaching a more advanced stage of embryonic development originated from oocytes that had CCs with a lower level of CCDFI. In our experiment, as 34.5% of the fertilized oocytes failed to become day 3 of development. Fertility clinics try to extend the culture of embryos until blastocyst stage [33]. Extending culture (to day 3–5) allows the embryologist to have a better assessment of embryo selection [34]. Oocytes that have been able to develop in vitro until day 5 are therefore regarded to be of higher quality, are more likely to result in pregnancy, and consequently have a low CCDFI value. Results from our study would support this hypothesis as the highest CCDFI values were those associated with the discarded embryos, and the lower CCDFI values were those that developed to d3 and d5 embryos. Using the TUNEL assay for assessment of DNA fragmentation, Bosco et al. [15] and Ruvolo et al. [16] reached a similar conclusion.

Results of the ROC curve (Fig. 3) to differentiate between oocytes whose embryos do not develop normally (rejected by embryologist at day 1) and those that develop to d3 or d5 revealed that CCDFI could be used to predict the fate of each oocyte; moreover, the cut-off for CCDFI was established as 21%; this cut-off value is in accordance with other studies of CC DNA fragmentation [14–16].

The results of our investigation suggest that based on a CCDFI threshold value of 24%, it was possible to discriminate the capacity of the oocyte to partake in successful syngamy with a 75.6% sensitivity and specificity of 65%. This information is likely to be important to both the clinician and embryologist as it could potentially reduce the need for unnecessary sperm injection and thereby allow more efficacy in the ICSI procedure if better quality oocytes can be selected in advance. In this study, we have also shown that CCDFI can be used as a predictor or reference marker when extending the in vitro development of embryos over time. Our results show that a CCDFI estimate not exceeding a 21% was able to identify oocytes that the fertility clinic would typically be classified as good quality and managed to reach maximum stage of development (3 or 5 days). In this way, this 21% DFI could be established as the maximum limit to identify a good quality oocyte. This would increase the efficiency of the implantation and, therefore, the rate of reproductive outcome in ART. Although CCDFI in this study was not helpful in predicting the morphological grade of embryos, we must bear in mind that some studies have shown that embryos with good morphology can still show chromosomal abnormalities [35].

Finally, we want to highlight that while the assessment of pooled CCs may indeed provide information about the general capacity of the female to produce oocytes of a certain quality (see Barcena et al., [17]), the individual analysis of single oocytes in preparation for ICSI provides more specific information about the quality of the individual oocyte. Large variation in CCDFI may exist when different oocytes from the same ovary are collected and this aspect deserves more investigation. Although our results are preliminary and require further validation using a larger sample size, there was nevertheless a clear relationship between CCDFI and oocyte/embryo development. In this study, we make the claim that a threshold CCDFI of less than 24% can be used to discriminate a good and compromises of a good oocyte quality with the levels of sensitivity and specificity above determined. Likewise, the ability to better predict ART success based on informed oocyte selection would reduce the need to collect and inject the number of oocytes required during ICSI protocols providing and additional criterion to select the best embryo to be transferred. Another area of possible application would be to use the test to help grade oocytes for oocyte cryo-banking; and only keep the best oocytes.
